# Development of Low-Cost In-House Assays for Quantitative Detection of HBsAg, HBeAg, and HBV DNA to Enhance Hepatitis B Virus Diagnostics and Antiviral Screening in Resource-Limited Settings

**DOI:** 10.3390/pathogens14030258

**Published:** 2025-03-05

**Authors:** Simmone D’souza, Layla Al-Yasiri, Annie Chen, Dan T. Boghici, Guido van Marle, Jennifer A. Corcoran, Trushar R. Patel, Carla S. Coffin

**Affiliations:** 1 Department of Microbiology, Immunology and Infectious Diseases, Cumming School of Medicine, University of Calgary, Calgary, AB T2N 2T8, Canada; simmone.dsouza@ucalgary.ca (S.D.); layla.alyasiri1@ucalgary.ca (L.A.-Y.); annie.chen2@ucalgary.ca (A.C.); dan.boghici@mail.mcgill.ca (D.T.B.); vanmarle@ucalgary.ca (G.v.M.); jennifer.corcoran@ucalgary.ca (J.A.C.); trushar.patel@uleth.ca (T.R.P.); 2 Department of Medicine, Cumming School of Medicine, University of Calgary, Calgary, AB T2N 2T8, Canada; 3 Department of Chemistry and Biochemistry, Alberta RNA Research and Training Institute, University of Lethbridge, Lethbridge, AB T1K 3M4, Canada

**Keywords:** hepatitis B virus (HBV), HBeAg, HBsAg, HBV DNA, qPCR, ELISA, column-free DNA isolation, tenofovir disoproxil fumarate (TDF), antiviral drug screening, resource-limited setting (RLS)

## Abstract

Globally, an estimated 254 million people are living with chronic hepatitis B virus (HBV) infection, yet only 10.5% have been diagnosed, underscoring the urgent need to expand testing to meet the World Health Organization’s HBV elimination targets by 2030. Many HBV diagnostic tests remain expensive and inaccessible in resource-limited settings. In this study, we demonstrate how individually sourced, commercially available reagents can be used to develop cost-effective in-house assays for total DNA isolation, HBV viral load quantification by (q)PCR, and qHBsAg and qHBeAg measurement using sandwich ELISA. These assays were validated using known HBV-positive and HBV-negative plasma samples (genotypes A–F) and HepAD38 cells treated with tenofovir disoproxil fumarate (TDF). DNA isolation using a commercial column-based kit was compared to a high-throughput, column-free method, allowing for HBV quantification from 50 µL of plasma with lower limits of detection (LLOD) of 1.8 × 10^3^ and 1.8 × 10^4^ HBV DNA copies IU/mL, respectively. Both commercial and in-house DNA isolation methods yielded comparable half-maximal effective concentration (EC_50_) values in TDF-treated HepAD38 cells. Additionally, in-house sandwich ELISA assays were developed for quantitative HBsAg and HBeAg detection, with LLOD values of 0.78 IU/mL and 0.38 PEI U/mL (Paul Ehrlich Institute), respectively. The in-house reagents for DNA isolation, molecular testing, and serological detection of HBV were estimated to be at least 10 times more cost-effective than commercially available kits, highlighting their potential for broader application in resource-limited regions.

## 1. Introduction

The hepatitis B virus (HBV), a member of the *Hepadnaviridae* family, is a blood-borne pathogen consisting of an enveloped partially double-stranded DNA genome. Despite the availability of an effective HBV vaccine, an estimated 254 million people globally are living with chronic hepatitis B virus (CHB) infection [1]. Approximately 880,000 deaths/year are directly attributed to HBV infection, as individuals living with CHB are at high risk of developing cirrhosis, liver cancer, and liver failure [1]. The World Health Organization (WHO) has set ambitious goals to eliminate viral hepatitis by 2030 through reducing new HBV infections by 90% and mortality by 65% [2], requiring investment and scaling-up of programs for HBV birth-dose vaccination, testing, and treatment to achieve these targets [3]. The most effective strategy to prevent chronic HBV infection has been the introduction of birth-dose vaccination, which has substantially reduced HBV infection rates in countries with high disease burden [4]. Unfortunately, there are no curative treatments for chronic HBV infection, as the currently used third-generation nucleos(t)ide analogs, such as entecavir and tenofovir, are unable to eliminate intranuclear HBV covalently closed circular DNA (cccDNA) or integrated DNA [5]. While current treatments cannot cure HBV, they significantly improve patient outcomes by effectively suppressing circulating HBV DNA levels in infected individuals and reducing the risk of mother-to-child transmission when administered in the third trimester [6]. Despite the effectiveness of vaccines and treatment options at preventing and treating chronic HBV infection, only, 10.5% of individuals living with CHB are aware of their infection status [1]. As such, HBV testing represents a major impediment to achieving the WHO’s elimination goals. To close this gap and identify those living with HBV, there must be greater accessibility to diagnostic tests that offer quick and easy ways to detect chronic hepatitis B (CHB) in individuals [7].

The hepatitis B virus has a complex life cycle and a dynamic natural history due to interplay between the virus and the host antiviral immune response [8]. The basis of viral persistence is due to the presence of intranuclear cccDNA within HBV-infected hepatocytes, as it serves as the template to produce all viral transcripts. CHB infection is diagnosed by the persistence of HBsAg in serum for greater than 6 months [9]. Several HBV serological markers (i.e., HBeAg, anti-HBe, anti-HBs, and HBV DNA) are used to monitor infection status and the need for antiviral therapy. Diagnostic tests rely on the detection of HBsAg and HBeAg using enzyme-linked immunosorbent assay (ELISA) and HBV DNA viral load using qPCR [10].

HBV disproportionately affects individuals within low- and middle-income countries or resource-limited settings (RLSs) [11,12]. Consequently, the high costs of standard commercial assays (i.e., HBsAg, HBeAg, and HBV DNA of ~$25, ~$65, and ~$325 USD/sample, respectively, Roche or Abbott Architect) are prohibitive for healthcare providers and researchers in RLSs [13,14,15]. Recent efforts have focused on the development of low-cost, rapid point-of-care (POC) tests to improve HBV screening (qualitative HBsAg and HBeAg) [13]. However, POC tests still require diagnostic laboratory confirmation by standard ELISA and assessment of other HBV markers to monitor disease status (i.e., quantitative or qualitative HBsAg, qualitative HBeAg, and quantitative HBV DNA levels). Moreover, in areas with limited access to HBV DNA testing, HBeAg positivity may be a surrogate indicator of high HBV replication [16].

Herein, we describe the development of in-house assays that are low-cost, quantitative, and highly sensitive in detecting HBsAg, HBeAg, and HBV DNA in plasma from HBV-positive individuals with HBV genotypes A–F and in tenofovir disoproxil fumarate (TDF)-treated HepAD38 cells. We also compare various DNA preparation methods (automated bead-based DNA isolation, silica-column-based, column-free, boil–spin, and extraction-free) for qPCR to determine the most cost- and time-effective option. Moreover, we also investigated the effects of the addition of the non-cytotoxic cell viability marker Alamar Blue on viral marker readouts to optimize an economical antiviral drug screening pipeline in HepAD38 cells. These economical methods may be suitable for use in both research and diagnostic labs, particularly in resource-limited settings. They could potentially serve as an intermediate solution, bridging the gap between point-of-care diagnostics and more costly commercial alternatives, thereby possibly enhancing HBV diagnosis and advancing research towards a cure in underserved areas.

## 2. Materials and Methods

### 2.1. Chronic HBV Patient Clinical Data and Sample Collection

All subjects were recruited from the University of Calgary Liver Unit and provided informed written consent to participate. A total of 2 HBV-negative donors and 35 known HBV+ patients who had provided a blood sample for biobanking were included in this study under an approved protocol by the conjoint health research ethics board, CHREB (Ethics ID: REB15-3137). Samples from patients with HBV genotypes A–F (as determined by a line probe assay; INNO-LiPA HBV Genotyping assay, Innogenetics N.V., Ghent, Belgium) with detectable levels of HBV DNA (>10 IU/mL or 50 copies/mL, Abbott Architect) were selected from the plasma biobank that had been stored long-term at −80 °C degrees. Standard clinical tests (HBsAg, HBeAg, and HBV DNA) using the Abbott Architect Platform were performed on all serum samples by the Alberta Precision Laboratories, Calgary, Alberta, Canada. An Abbott RealTime HBV Viral Load assay was used for the quantification of HBV DNA, An Abbott HBsAg Quantitative assay was used for the quantification of HBsAg, and an ARCHITECT HBeAg assay was used for the qualitative detection of HBeAg.

### 2.2. Quantitative In-House Sandwich ELISA for Detection of HBsAg and HBeAg

It should be noted that Fitzgerald industries has been acquired by Biosynth*®*, hence all future antibody purchases should be purchased through Biosynth*®* with the same catalog numbers listed below. In this study, 96-Well High-Binding Microtiter Plates (CLS9018, Millipore Sigma, Burlington, MA, USA) were coated overnight at 4 °C with 100 µL of (1 ng/µL) α-HBsAg mouse monoclonal antibody (10-H05H, Fitzgerald, Acton, MA, USA) or α-HBeAg mouse monoclonal antibody (10-H10M, Fitzgerald, Acton, MA, USA) diluted in coating buffer (0.17% Na_2_CO_3_, 0.29% NaHCO_3_, pH 9.6). Following incubation with the primary antibody, the plates were washed once with 100 µL of PBST (0.2% Tween-20 in PBS), and each well was incubated with 150 µL of blocking buffer (2% BSA in PBS) at 37 °C for 1 h on a shaking platform (75 rpm). Following blocking, the plates were washed four times with 100 µL of PBST. Protein standards ranging from 0.78–50 ng/mL for HBsAg protein standard (C-CP2019R, Fitzgerald, Acton, MA, USA) and 0.39–50 ng/mL for HBeAg protein standard (30-AH18, Fitzgerald, Acton, MA, USA) were prepared in blocking buffer, and 100 µL of each standard was added to the plate in triplicate. For HepAD38 cells seeded in 96-well plates, 1:2 dilution was performed in blocking buffer, and 100 µL of the diluted supernatant was added per well. The plasma from each individual patient was diluted 1:10–1:1000 (e.g., 10 µL of plasma in 90 µL of blocking buffer), and 100 µL of the dilution was added to the plate in triplicate. Following the addition of the standards and samples, the plates were incubated at 37 °C for 1 h on a shaking platform (75 rpm) or at 4 °C overnight and then washed four times with 100 µL of PBST. Next, 100 µL of the appropriate HRP-conjugated detection antibody diluted in blocking buffer was added to each well, either α-HBsAg goat polyclonal antibody (70-HG15S, Fitzgerald, Acton, MA, USA) or α-HBeAg mouse monoclonal secondary antibody (61-H10K, Fitzgerald, Acton, MA, USA). The plates were incubated at 37 °C for 1 h in a shaking incubator (75 rpm), washed four times with 100 µL of PBST, and then incubated in the dark with 100 µL of the TMB (3,3′,5,5′-tetramethylbenzidine) substrate (00-2023, Thermo Fisher Scientific, Waltham, MA, USA). Following a 15 min incubation with TMB at room temperature on a shaking platform, 100 µL of freshly prepared 1 M hydrogen chloride (HCl) was added to each well, and the plate was visualized at 450 nm using a plate reader (SpectraMax i3x, Molecular Devices, San Jose, CA, USA). The α-HBsAg goat polyclonal detection antibody used in the sandwich ELISA (70-HG15S, Fitzgerald, Acton, MA, USA) was prepared in advance by conjugating HRP at 4:1 (antibody-to-HRP ratio) using a commercial kit (LNK002P, Bio-Rad, Hercules, CA, USA).

### 2.3. HBV Cell Culture and Viability Assessment

HepAD38 Cells (gift from Dr. Christoph Seeger, Fox Chase Cancer Center, Philadelphia, PA, USA) seeded in 24-well or 96-well plates were maintained in a humidified incubator at 37 °C with 5% CO_2_, with all reagents supplied by Thermo Fisher Scientific (Waltham, MA, USA): Dulbecco’s Modified Eagle Medium (DMEM) with Glutamax and pyruvate (10569044) supplemented with 10% FBS (12484028), 1× MEM non-essential amino acids (11140050), 100 IU/mL penicillin/100 µg/mL streptomycin (15140122), and 400 µg/mL geneticin (10131035). Tenofovir disoproxil fumarate (TDF, SML1794, Sigma-Aldrich, St. Louis, MO, USA) was reconstituted to 1 mg/mL in PBS and serially diluted (50 µM to 0.0015 µM) for treatment of HepAD38 cells. Media and TDF were replenished every 3 days, and cells were harvested 14 days post-treatment. On day 14 post-treatment, 10 µL/well of Alamar Blue (DAL1025, Thermo Fisher Scientific, Waltham, MA, USA) was added to each 96-well plate and incubated for 2 h at 37 °C in a humidified incubator with 5% CO_2_ prior to absorbance reading at 570 nm and 600 nm (SpectraMax i3×, Molecular Devices, San Jose, CA, USA). Cell viability was calculated using standard methods [17].

### 2.4. DNA Isolation from Plasma or Cell Culture Supernatant Using Column-Based, Column-Free, or Boil–Spin Methods

Total DNA was isolated using a commercial silica-column-based method (i.e., DNeasy Blood and Tissue Kit, Qiagen, Hilden, Germany) from 50 µL of patient plasma or HepAD38 cell culture supernatant. The cell culture supernatant collected from the 24- or 96-well plates was transferred into 1.5 mL tubes or 96-well PCR plate (Alamar-Blue-treated and non-Alamar-Blue-treated) and spun at 1000× *g* for 5 min to pellet cellular debris prior to extraction, according to the manufacturer’s protocols, and eluted in 100 µL of DNase-free water.

In parallel, total DNA was isolated from 50 µL of plasma or HepAD38 cells using an in-house column-free DNA extraction method. In brief, cells were washed once with PBS, and cell lysates were prepared by incubating with isolation buffer (200 µL for 24-well plates and 50 µL for 96-well plates of 100 mM Tris pH 8.0, 5 mM EDTA, 0.2% SDS, 200 mM NaCl, 0.25% NP-40, and 200 ug/mL Proteinase K; BioBasic, Markham, ON, Canada) for 1 h at room temperature on a shaking platform. Following lysis buffer incubation, an equal volume of cold 100% isopropanol was added. The mixture from the 24-well plates was transferred into 1.5 mL tubes, while the 96-well cell culture plate was transferred directly to another 96-well PCR plate (82006-650, VWR, Radnor, PA, USA). Both samples were spun at 3260× *g* for 10 min to precipitate the DNA, the supernatant was discarded, the pellet was dried at room temperature, and samples from the 24-well plate and 96-well plate were resuspended in 100 µL and 50 µL, respectively, of DNase-free water before qPCR quantification. Similarly, 50 µL of plasma from patients was subject to the column-free DNA isolation method with modifications (150 µL of lysis buffer with 1 h room-temperature incubation followed by addition of 200 µL of 100% isopropanol) and spun at 18,000× *g* for 2 min to precipitate the DNA. Following room-temperature drying, the DNA pellet was resuspended in 50 µL of DNase-free water and spun again at 5000× *g* for 2 min to pellet any non-water-soluble aggregates. The resulting supernatant was transferred to a fresh 1.5 mL tube for HBV DNA quantification. To determine the extraction efficiency, we spiked 50 µL of healthy plasma with known copies of HBV DNA (10–10^7^ copies/µL; 1.8 × 10^3^–1.8 × 10^9^ IU/mL) and extracted the DNA using either the Qiagen silica-column-based kit or column-free DNA isolation, as described above, and eluted the samples in 50 µL of DNase-free water.

The boil–spin method described by Vanhomwegen et al. (2021) was also utilized as another DNA extraction platform to be used in RLSs [18]. In brief, 50 µL of plasma was diluted 2-fold with 50 µL of nuclease-free water, incubated at 95 °C for 5 min, and then spun down at 12,000× *g* for 10 min.

### 2.5. Quantification of Total HBV DNA

HBV DNA qPCR was performed using the Biorad C1000 Touch CFX96 Real Time System with iTaq Universal SYBR Green Master Mix (Bio-Rad Laboratories, Hercules, CA, USA). HBV standard curves were developed using the HBV 1.3 mer genotype D plasmid (gift from Dr. Wang Shick Ryu, Addgene# 65459) [19]. Preparation of 10^9^ copies/copies/µL stock was prepared using the DNA copy number and dilution calculator on the ThermoFisher Scientific website. A custom DNA fragment was chosen, and the genome length was adjusted to the plasmid size, which was 6821 bp, and the stock concentration was adjusted to the concentration of the miniprepped plasmid, and dilution was set to 10^9^ copies/µL. Ten-fold serial dilutions in DNase-free water were carried out to achieve the desired DNA copy number of 10–10^8^ copies/µL.

For all qPCRs performed, 10 µL of reaction mixture was used per well, containing 1 µL of template (either DNA extracted from cells or plasma or cell-free supernatant) and 0.5 µM of forward (HBV 2270F) and reverse (HBV 2392R) primers, using previously published PCR primer sequences [20] but with modified cycling conditions (95 °C for 10 min, followed by 45 cycles of 95 °C for 5 s and 60 °C for 30 s).

### 2.6. Z′ Score Calculation

The paper published by Zhang et al. (1999) introduced the Z′ factor as a statistical measure to evaluate the quality of high-throughput screening (HTS) assays [21]. It assesses the separation between positive and negative controls, helping to determine assay robustness and reliability.

$$Z^{\prime }=1-\frac{3\left(\sigma_{p}+\sigma_{n}\right)}{|\mu_{p}-\mu_{n}|}$$

where:

σₚ = Standard deviation of the positive control;

σₙ = Standard deviation of the negative control;

μₚ = Mean of the positive control;

μₙ = Mean of the negative control.

Interpretation:

Z′ > 0.5: Excellent assay;

0 < Z′ ≤ 0.5: Acceptable assay;

Z′ ≤ 0: Poor assay.

For the ELISA assays, the negative control was the BSA + 1° + 2° antibodies, and the positive control was the highest concentration of protein used in the standard curve.

For the qPCR assays used in the TDF screening, cells treated with PBS served as the negative control, while the positive control was the highest amount of TDF used without causing cell toxicity.

### 2.7. Cost Analysis

The costs of the reagents used in the above assays were determined by obtaining prices in Canadian dollars (CAD) from the suppliers. Details of the cost breakdown can be found in the Supplementary Document (Tables S1–S4). The cost of labor and laboratory equipment was not included, as this varies geographically.

## 3. Results

### 3.1. Development of an In-House Sandwich ELISA for the Detection of HBsAg and HBeAg

We aimed to develop a low-cost in-house ELISA to quantify HBsAg and HBeAg to be used to screen plasma samples from patients with HBV infection or in antiviral drug screening studies. To develop a quantitative sandwich ELISA for the detection of HBsAg, we first needed to determine an antibody combination that would be cross-reactive to HBsAg genotypes A–F. Several commercially available antibodies (Supplemental Figures S1 and S2) were tested against the genotype C HBsAg protein standard and genotype D HBV-positive supernatant derived from HepAD38 cells in a dot-blot format to determine the best combination to be used for a sandwich ELISA. We found that the ideal combination was a mouse monoclonal antibody against HBsAg (Fitzgerald, 10-H05H) and goat polyclonal HBsAg antibody (Fitzgerald, 70-HG15S); however, since neither antibody is available commercially with a horse-radish peroxidase (HRP) tag, advanced antibody preparation was required using the 70-HG15S antibody with a Lynx Rapid HRP Antibody Conjugation Kit (LNK002P, BioRad). For HBeAg detection, antibodies were tested from Fitzgerald industries; the primary antibody was α-HBeAg mouse monoclonal antibody (Fitzgerald, 10-H10M), and the secondary antibody was α-HBeAg mouse monoclonal antibody (Fitzgerald, 30-AH18).

**Figure 1:**
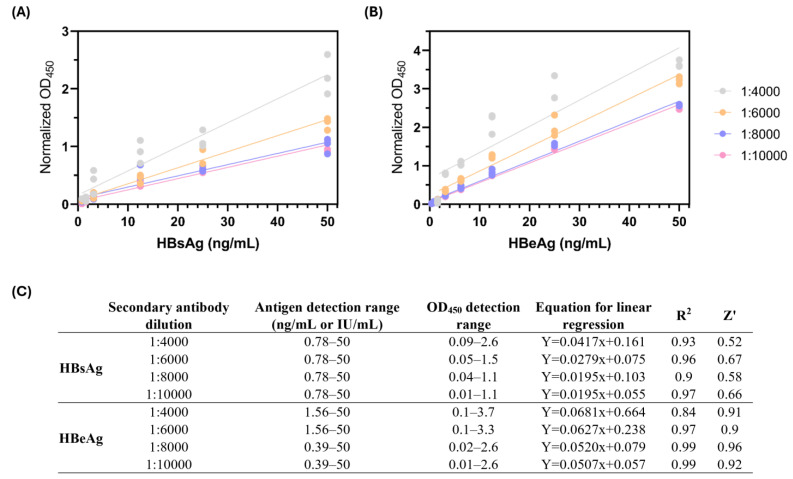
Optimization of in-house quantitative sandwich ELISA for detection of HBsAg and HBeAg. Standard curve depicting the optimal dilution of detection antibody for the detection of (**A**) HBsAg and (**B**) HBeAg. Primary antibodies used for antigen capture were mouse monoclonal α-HBsAg (Fitzgerald, 10-H05H) and mouse monoclonal α-HBeAg (Fitzgerald, 10-H10M). Detection was achieved using HRP-conjugated α-HBsAg goat polyclonal antibody (Fitzgerald, 70-HG15S) and α-HBeAg mouse monoclonal antibody (Fitzgerald, 61-H10K). OD₄₅₀ values were normalized against BSA control wells. The assay was optimized by adjusting antibody concentrations to achieve a strong signal-to-noise ratio while maintaining linearity. Data were normalized by subtracting the optical density reading at 450 nanometers (OD_450nm_) from bovine serum albumin (BSA) control wells. (**C**) Summary table of ELISA data for HBeAg and HBsAg. Assay quality was assessed based on linearity of the standard curve R^2^ and Z′ factor. Data from 3 biological replicates.

Given that most plate readers have an upper absorbance limit of 3 OD (optical density), we first determined the highest secondary antibody dilution with the broadest antigen detection for the detection (within the range of 1–2.5 OD) of HBsAg and HBeAg (Figure 1A,B). The strength of these assays was evaluated by the Z′ score, where a score of 0.5 ≤ Z′ < 1 indicates a “high-quality” assay [21]. Based on these parameters, a 1:6000 dilution of the HBsAg detection antibody and 1:8000 dilution of the HBeAg detection antibody (equivalent of 1 ng/mL final concentration) were selected (Figure 1C) for further experiments. This provided an assay detection range of 0.78–50 ng/mL for HBsAg and 0.39–50 ng/mL for HBeAg.

**Figure 2:**
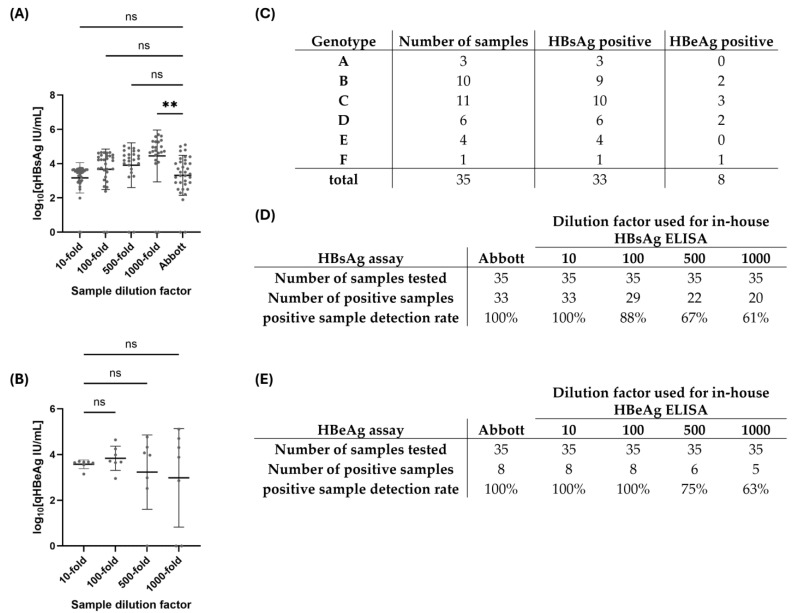
Comparison of quantitative in-house ELISA assays for HBsAg (**A**) and HBeAg (**B**) detection in plasma samples from HBV-infected individuals (genotypes A–F), evaluated against a commercial ELISA (Abbott Architect). Dilution series (10-, 100-, 500-, 1000-fold) were conducted to determine the minimum plasma volume required for reliable quantification of HBsAg and HBeAg. Statistical analysis via one-way ANOVA with Tukey’s HSD post hoc test revealed significant differences (**) at 1000-fold dilution between groups (*p* < 0.05), and no significant (ns) differences between other dilution series. Data are presented as mean ± SD from three technical replicates per sample. (**C**) Summary of 35 samples tested, detailing HBsAg and HBeAg positivity rates across genotypes (A–F), as determined by the Abbott Architect assay. A 10-fold plasma dilution (10 µL in 100 µL of blocking buffer) reliably quantified (**D**) HBsAg and (**E**) HBeAg. Notably, HBeAg levels in samples tested from genotypes A and E were below the lower limit of detection (<LLOD) in both the in-house and Abbott Architect assays.

According to the manufacturer’s details, both the capture and detection antibodies used in the quantitative in-house HBsAg ELISA should be cross-reactive with all four subtypes of HBsAg—adr, adw, ayr, and ayw [22]. To determine if our in-house ELISAs could recognize HBV genotypes A–F, plasma samples were obtained from HBV-positive patients (Figure 2). Using blinded reference testing, the following criteria were used to select the plasma samples: (i) HBsAg-positive with known HBV genotypes (A–F); (ii) either HBeAg-positive or -negative plasma, and (iii) detectable HBV DNA in plasma. In total, 35 patients were selected, representing each genotype, and 2 HBV-negative human plasma samples were used to evaluate signal specificity. Varying plasma dilutions from the HBV+ plasma were tested to determine the optimal concentration that would provide reliable quantification. For the detection of HBsAg by ELISA (Figure 2A) and HBeAg (Figure 2B), sample dilutions between 1:10–1:1000 were tested and compared to the commercial assay (Abbott Architect platform). For the HBeAg ELISA (Figure 2B), our biobank did not have any HBeAg+ samples from genotype A or genotype E, and all the tested samples for these genotypes were below lower limit of detection (<LLOD), which was consistent with clinical testing (Abbott). Since the Abbott assay for HBeAg is qualitative, it is not plotted in Figure 2B, and the clinical findings are summarized in Table 1. A summary of the genotypes tested and HBsAg or HBeAg positivity, as determined by the Abbott Architect platform, is summarized in Figure 2C, while the results from the dilution series (1:10 to 1:1000) are described for HBsAg in Figure 2D and HBeAg in Figure 2E. The 1:10 dilution most consistently tested positive for HBsAg, while 1:10–1:100 worked for HBeAg+ individuals. Conversions of 1 ng/mL to 1 IU/mL for HBsAg and 1 ng/mL to 1 PEI U/mL for HBeAg were used to convert the data to international unit nomenclature [15,23,24].

### 3.2. Validation of In-House qPCR and Column-Free DNA Isolation Methods for Subsequent HBV DNA Quantification

**Figure 3:**
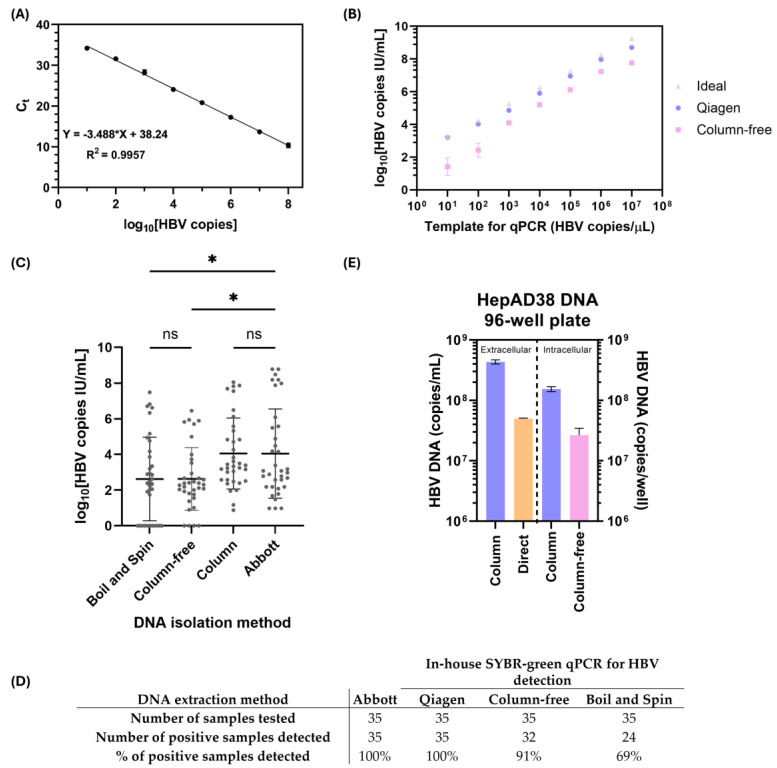
Summary of in-house assay optimization for HBV DNA quantification. (**A**) Detection of HBV DNA from 10–10^8^ copies, plasmid dilutions with standard curve. (**B**) Comparison of column-based and column-free DNA isolation methods for quantification of HBV DNA from healthy plasma spiked with 10–10^7^ copies/µL HBV plasmid DNA. HBV copies/µL were converted to log_10_ IU/mL, where 1 IU/mL = 5.26 HBV DNA copies, and plotted to determine the dynamic range of extraction. (**C**) Total DNA isolated from 50 µL of patient plasma using commercial silica- column-based DNA purification, in-house column-free DNA isolation, or boil–spin method, and HBV was quantified using an in-house SYBR-Green-based qPCR assay and compared to commercial diagnostic assay (Abbott Architect platform). (**D**) Summary table of findings from samples tested in panel (**C**). Statistical analysis via one-way ANOVA with Tukey’s HSD post hoc test revealed significant differences (*) between column-free and boil-spin method when compared to the Abbott assay (*p* < 0.05), and no significant (ns) differences between Abbott and column method. (**E**) HBV DNA quantification of HepAD38 cells seeded in 96-well plates. Extracellular (supernatant) total DNA was first isolated using a silica column, or HBV DNA was directly quantified by qPCR. Intracellular total DNA was either extracted using silica-column-based or column-free methods before HBV DNA qPCR. Data are plotted as the mean and standard deviation. Three biological replicates were performed for panels (**A**,**B**,**E**), and three technical replicates were performed for each patient sample in panel (**C**).

Due to the high costs associated with commercially available diagnostic tests for HBV DNA, we aimed to develop a more economical qPCR setup. Using a 1.3 mer HBV plasmid (gifted by Dr. Wang Shick Ryu) and primers designed to detect all genotypes of HBV (2270F, 2392R) [20], we optimized an in-house qPCR assay (Figure 3A) that has a detection range of 10–10^8^ HBV copies. Research labs that perform HBV viral load testing in clinical and non-clinical samples routinely use non-automated, silica-column-based kits for total DNA isolation (i.e., Qiagen DNeasy Blood and Tissue, Hilden, Germany) [14,25]. Silica-based columns provide high-quality DNA for qPCR but are impractical for high-throughput screening due to the limitation of the number of tubes that fit in a benchtop centrifuge (max 24), higher costs, and a laborious protocol with several centrifugation and washing steps. As such, we wanted to compare the DNA quality obtained from silica-based columns to a column-free DNA preparation method based on a protocol previously described for mammalian cells [26]. The manufacturer’s insert from the silica-column-based Qiagen DNeasy Blood and Tissue kit recommends extracting DNA from 50–200 µL of plasma, with an elution volume of 50–150 µL. To determine the extraction efficiency of the silica-column-based kit and column-free DNA isolation, we spiked 50 µL of healthy plasma with known copies of the 1.3 mer HBV plasmid DNA and tested 1 µL of the extracted samples by qPCR (Figure 3B). The lower limits of detection for the Qiagen DNeasy Blood and Tissue kit and the column-free method were 10^3^ HBV copies/mL (1.8 × 10^3^ IU/mL) and 10^4^ HBV copies/mL (1.8 × 10^4^ IU/mL), respectively (Figure 3B).

We applied the silica-column-based and column-free DNA isolation methods to patient plasma to assess how the in-house qPCR and DNA isolation methods compared to the Abbott Architect system. Total DNA was isolated from 50 µL of plasma (Figure 3C), since increasing the starting volume to 200 µL did not increase the dynamic range of detection or affect the LLOD, likely due to the higher presence of contaminants interfering with qPCR. Overall, the silica-column-based DNA isolation provided comparable viral load quantifications to the commercial Abbott assay, but the column-free DNA isolation method showed ~1 logs lower DNA levels (Figure 3B,C) [14,15]. Another DNA-based assay that has been proposed for use in RLSs is the boil–spin DNA isolation method. The boil–spin method provided similar results to the column-free DNA isolation method (Figure 3C), but it had a lower % positive sample detection rate (Figure 3D).

Although the Abbott Architect platform is standard for high-throughput drug screening in cell cultures, it remains costly and inaccessible to researchers with limited resources. The silica-column-based method for DNA isolation is cheaper, but it is not high-throughput, despite providing a good-quality DNA template. To increase the throughput of potential drug screening libraries that impact HBV DNA secretion or production, we investigated whether it was feasible to perform direct HBV DNA quantification from the supernatant of HepAD38 cells or column-free DNA isolation from cells. We tested various HBV DNA preparation methods (Figure 3E) in HepAD38 cells seeded in 96-well plates. We determined that HBV could be directly quantified from extracellular cell-free supernatant and provided a ~1 log lower DNA yield than DNA prepared using the silica-based column. Similarly, for intracellular HBV quantification, the column-free DNA preparation resulted in ~1 log lower HBV DNA quantified compared to the silica column.

### 3.3. Comparison of Silica-Column-Based Nucleic Acid Purification Kit to In-House DNA Isolation Protocols for Determination of Tenofovir Disoproxil Fumarate Half-Maximal Effective Concentration (EC_50_)

**Figure 4:**
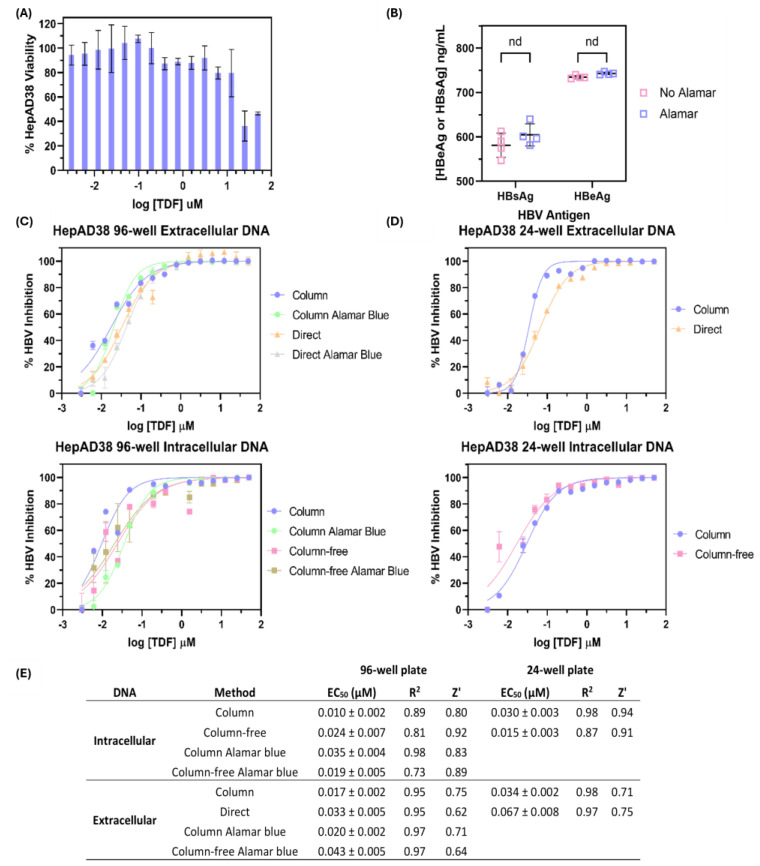
Comparison of various DNA isolation methods for determining the EC_50_ of TDF in HepAD38 cells. HepAD38 cells were seeded in 24-well plates or 96-well plates and treated with TDF for 14 days with media and drug replenishment every 72 h. (**A**) To determine the toxicity of TDF, HepAD38 cells in the 96-well plate format were incubated with Alamar Blue for 2 h prior to reading the absorbance at 560 nm and 600 nm. Data were normalized according to the manufacturer’s directions to determine cell viability. (**B**) HBeAg and HBsAg could be quantified from the supernatant of 96-well HepAD38 cells treated with Alamar Blue. Extracellular (cell supernatant, top panel) and intracellular (bottom panel) HBV DNA were determined in (**C**) 96-well and (**D**) 24-well plate formats and plotted as % HBV inhibition against log [TDF] to determine the EC_50_. To investigate if Alamar Blue negatively impacts EC_50_ determination, HepAD38 cells from the 96-well plate treated with Alamar Blue were also subjected to DNA isolation and quantification. (**E**) Summary of TDF EC_50_ values obtained from the treatment of HepAD38 cells in either a 24- or 96-well format. Assay Z′ factors are given for each DNA isolation method, and all displayed a high signal-to-background ratio. For each data point, four biological replicates were performed and plotted to include the mean ± standard deviation. A *t*-test was performed for panel (**B**) where n.d. indicates no statistical difference.

Antiviral drug screening studies targeting various aspects of the HBV life cycle require reliable tools to measure cell viability, HBsAg, HBeAg, and HBV DNA. As a proof of concept, we used a known potent anti-HBV reverse transcriptase inhibitor, TDF, to treat HepAD38 cells in 24-well and 96-well plates. Drug cytotoxicity was measured using Alamar Blue, as this reagent is non-cytotoxic and provides information on cell metabolism while being relatively cheap/easy to use [27]. Based on the Alamar Blue cell viability assay, TDF appeared to be toxic to HepAD38 cells at concentrations higher than 12.5 µM (Figure 4A). The addition of Alamar Blue to the cells did not impact the measurement of HBsAg/HBeAg (Figure 4B) and HBV DNA (Figure 4C).

Total DNA isolation using silica-based columns is impractical in large drug screening libraries due to the low-throughput, albeit yielding reliable, high-quality template DNA. Thus, we compared the silica-column-based DNA extraction method to the column-free DNA isolation method to determine the difference in the EC_50_ of TDF. We also investigated if the EC_50_ determination of TDF was different in the 96-well plate (Figure 4C) vs. the 24-well plate (Figure 4D). The results obtained from the column-based and column-free DNA isolation methods of HepAD38 cells treated with TDF in the 24-well plate, 96-well plate, and Alamar-Blue-treated 96-well plate all fell within the EC_50_ range of 8 nM-75 nM range (Figure 4E), and this was consistent with the literature value of 2–200 nM [28,29]. The Z′ score for all extraction methods (0.5 ≤ Z′ < 1) indicated a “high-quality” assay, allowing for drug screening in the 96-well plate format [21]. Despite this 1 log variation in HBV quantification, this did not negatively impact the EC_50_ determination of TDF. As such, using a column-free method can be beneficial to researchers in some settings, especially for large-library antiviral compound screening, as technician time can be saved. The addition of a cell viability agent (Alamar Blue) did not impact quantification of HBV DNA, HBsAg, and HBeAg. Although HBeAg and HBsAg could be measured in the HepAD38 cells (Figure 4B), it was not assessed in the TDF-treated cells, since they are not affected by short-term reverse transcriptase inhibitor therapy.

## 4. Discussion

In low- and middle-income countries, numerous barriers exist for the diagnosis of viral hepatitis in a timely and accurate manner. One of the main factors is cost (equipment and reagents) and access to well-trained staff with a laboratory equipped to perform diagnostic testing [13]. To bridge these barriers, accurate and cost-effective point-of-care testing is required. The commercial sandwich ELISAs used for measuring HBsAg and HBeAg and qPCR used for measuring HBV DNA are standard and routine diagnostic assays performed to confirm HBV infection and monitor virus status in patients. However, these standard diagnostic clinical assays have a high estimated per-sample cost and pose a significant impediment to global screening efforts for HBV (Table 2) [13]. The most cost-effective screening method at the population level is the implementation of point-of-care lateral flow tests, which use a needle prick to obtain 50–60 µL of blood to use for testing of HBeAg and HBsAg [13]. The lateral flow assays used for the detection of HBsAg are highly sensitive, but the HBeAg assay is less sensitive [30,31]. In RLSs, HBeAg positivity has been suggested as a surrogate marker for high levels of HBV replication, where qPCR for HBV DNA load may be unavailable [32], and for informing HBV management (i.e., treating women during late pregnancy with high viral loads to prevent mother-to-child HBV transmission) [16].

Access to cheaper, but accurate, quantitative tests will benefit the clinical management of HBV and researchers testing new therapeutics in resource-limited settings. Qualitative tests are important and are still routinely used in clinics to determine HBsAg and HBeAg positivity and for the diagnosis of HBV. However, emerging studies are looking towards the usage of quantitative HBsAg and HBeAg as important markers to monitor disease progression, treatment decisions, treatment outcomes, and risk stratification for liver disease [36,37]. In this study, we showed that our in-house-developed platforms for quantitative HBsAg, HBeAg, and HBV DNA detection had a comparable cost/sample to point-of-care tests (Table 2). Approximately 70 µL of patient plasma is required to complete all our in-house-developed assays (i.e., quantitative HBsAg, HBeAg, and HBV DNA), which is a similar sample volume required for point-of-care lateral flow assays. Therefore, obtaining samples from a needle prick could be sufficient for the assessment of key quantitative HBV markers in addition to point-of-care lateral flow tests.

The Paul Ehrlich Institute (PEI) has also established an international standard for HBeAg detection, which is reported in PEI U/mL and was validated by comparing the testing of serum samples for HBeAg by 14 different national laboratories [38]. Despite having an international standard for quantitative HBeAg detection, most commercial platforms, including the Abbott Architect platform used by clinical labs, are qualitative in nature (positive or negative, LLOD 0.5 PEI U/mL). The HBsAg and HBeAg concentrations in ng/mL from our assay were converted to international units (IU)/mL using the accepted conversion of 1 IU/mL = 1 ng/mL HBsAg or 1 PEI U/mL = 1 ng/mL HBeAg [15,23,24]. Using this conversion, we determined that our LLOD values for the in-house sandwich ELISAs were 0.78 IU/mL for HBsAg and 0.38 PEI U/mL for HBeAg. As such, our in-house HBsAg ELISA was less sensitive when compared to the Abbott Architect platform (LLOD, 0.041 IU/mL) and slightly more sensitive for HBeAg detection (LLOD, 0.5 PEI U/mL).

We demonstrated that DNA isolation can be performed from 50 µL plasma using a commercial silica-column-based (LLOD, 1.8 × 10^3^ IU/mL) or column-free DNA isolation (LLOD 1.8 × 10^4^ IU/mL) method and can be accurately quantified using an in-house qPCR (LLOD, 10 HBV copies) protocol at a fraction of the cost of routine laboratory testing procedures (Table 2). The decision to use smaller starting sample volumes (50 µL vs. the 500 µL used by the Abbott Architect platform) was driven by our intent to determine the minimal sample volume that could still provide reliable quantification and to align with the maximum sample volume capacities of the HBV DNeasy Blood and Tissue Kit.

In cases where more precise DNA quantification is necessary, such as monitoring patients with low-to-moderate HBV DNA levels (i.e., confirming adherence to treatment and/or potential viral breakthrough on suppressive antiviral therapy), the silica-column-based DNA isolation method is recommended due to its lower limit of quantification (LLOD of 1.8 × 10^3^ IU/mL). We acknowledge that the column-free or boil–spin DNA isolation methods for plasma samples, although cheaper and more capable of high-throughput screening without automation, are less sensitive when compared to the silica-column-based DNA isolation method. Despite these limitations, both DNA isolation methods can still be valuable in certain scenarios. For instance, in RLSs, where the primary goal is to categorize patients broadly into those with high HBV DNA levels versus moderate HBV DNA levels, both methods could offer a cost-effective solution, especially when more costly resources are unavailable. Expert guidelines recommend nucleos(t)ide analog treatment during pregnancy if the maternal viral load is >200,000 IU/mL [39,40]. Treatment is also recommended if HBV DNA is >2000 IU/mL in persons with persistently elevated liver transaminases. In both of those clinical scenarios, an assay with an LLOD of 10^4^ IU/mL could be practically applied either to treat individuals to reduce mother-to-child transmission and others who are at risk of progressive liver disease. In comparing the column-free method to the boil–spin method, 69% vs. 91% of the samples tested positive (Figure 2), indicating that although both assays had a comparable LLOD, the column-free method might be more reliable for DNA isolation from moderately high-HBV-DNA samples [18]. Despite the lower assay sensitivity of the boil–spin method, the simplicity and cost-effectiveness of the method might be preferable to the column-free method for field-based applications. Other useful applications of the column-free DNA isolation method can be applied to resource-limited research settings for screening cell lines infected with HBV due to the high levels of viral DNA typically present (Figure 4).

Finally, for researchers involved in anti-HBV therapeutic development, we showed that HBsAg, HBeAg, and HBV DNA could be directly quantified using HBV-cell-free supernatant and that the column-based, column-free, and extraction free methods provided similar EC_50_ values for TDF. Since all three methods provided similar EC_50_ values, our in-house methods can likely be used for the evaluation of other HBV antivirals at a fraction of the cost of commercially available kits. Moreover, prior treatment of the HBV cell cultures with viability agents (Alamar Blue) did not affect the quantification of these HBV replication markers (Figure 4).

We acknowledge that there are several limitations to our study. Due to the limited number of patients genotyped in our lab, we tested our methods on a small number of patient samples (*n* = 35), representing genotypes A–F. We acknowledge that further evaluation on a larger cohort, including samples with low HBsAg, HBV DNA, and HBeAg negativity, would provide more insight into the lower detection limits of our assays and allow for more comprehensive comparisons with commercially available platforms and point-of-care testing platforms. This study did not account for the fact that HBeAg loss may reflect either improved host immune control of HBV—often indicated by anti-HBe seroconversion—or the development of immune escape mutations in the HBV precore or core (C) gene, which reduce or abolish HBeAg production [32]. These factors may impact the reliability of HBeAg as a surrogate marker for cccDNA activity. Moreover, methodologically, we did not test our platform using a robotic system due to lack of access. The inclusion of automated liquid handling systems is an important consideration to have, especially in regions with high endemicity, as these assays will need to be adapted to high-throughput testing, either with robotics or utilizing point-of-care diagnostics.

For the cost analysis of the in-house assays, only the reagent cost was considered for the samples processed, and accessibility to these reagents and costs may vary across the globe. Additionally, the cost of technicians and laboratory equipment was not factored in, as labor costs vary geographically, and equipment costs can vary depending on the product chosen. Unfortunately, we were unable to obtain the costs of the reagents from commercial vendors. In addition, our literature review did not find any publications disclosing the reagent costs associated with most proprietary testing platforms. Due to this limitation, we relied on one publication by Gani et al. (2019), where they utilized costs provided by a single commercial vendor, which were inclusive of reagent costs and labor costs [14,15].

In summary, the current work contributes to the ongoing development of more cost-effective methods used for HBV diagnostics, research, and drug screening to ultimately achieve the WHO’s 2030 elimination goals for hepatitis B. The development of a low-cost assay is only the first step in increasing access to essential public health laboratory tests for hepatitis B virus; however, we also acknowledge that this addresses only part of the challenge involved in HBV testing [40]. While these assays make testing more affordable, several other critical factors must be addressed to ensure the effective and widespread implementation of HBV testing. Access to equipment and infrastructure is a significant barrier, as many regions, especially those with limited resources, may lack the necessary infrastructure to conduct these tests. Furthermore, efficient distribution systems are essential to ensure that both the assays and the equipment reach remote and underserved areas promptly. Although all the reagents discussed in this paper are available for commercial purchase, supply chain issues may arise, and production of these reagents must also be scaled up to meet global demand. These issues require investment in manufacturing capabilities and supply chain logistics. Regulatory approval processes can vary widely by country, posing another layer of complexity, potential delays, and cost increases. Finally, the training of healthcare personnel is crucial; even with the best tools and technology, accurate and reliable HBV testing depends on skilled operators who can perform the tests correctly and interpret the results accurately. Addressing these challenges holistically is essential to creating an effective, sustainable, and equitable HBV testing program.

Fortunately, there has been significant investment in enhancing diagnostic services for HIV testing. This effort has been led by both governmental organizations, such as the World Health Organization, and non-governmental organizations, such as the Gates Foundation. Similarly, during the SARS-CoV-2 pandemic, substantial resources were dedicated to increasing laboratory capacity with equipment and supplies. Leveraging and strengthening this already established infrastructure and resources would make it feasible to implement cost-effective hepatitis B tests without diverting essential resources or trained personnel away from HIV and SARS-CoV-2 testing [41]. However, there remains a lack of awareness about the burden of hepatitis B virus infection, as well as stigma, discrimination, and reluctance towards voluntary self-testing [42]. To address these challenges, greater investment by governmental agencies and foundations, increased public–private partnerships, public advocacy, and leadership are needed to prioritize training key laboratory personnel and ensuring access to quality hepatitis B testing [40].

## Figures and Tables

**Table 1:** Summary of data comparing results of testing plasma samples from 35 individual patients with HBV genotype A–F infection using in-house column, column-free, or boil–spin DNA isolation methods prior to HBV DNA quantification by in-house SYBR-Green-based qPCR and quantitative in-house HBsAg or HBeAg sandwich ELISA versus clinical diagnostic assays (commercial PCR and ELISA, Abbott Architect). n.d.: not detected.

		HBV DNA Copies (log_10_IU/mL)				Quantitative HBsAg ELISA (log_10_IU/mL)		HBeAg ELISA Qualitative (-/+) or Quantitative (log_10_IU/mL)	
Patient ID	HBV Genotype	Abbott Architect DNA Isolation and qPCR	Silica Column DNA Isolation and In-House qPCR	Column-Free DNA Isolation and In-House qPCR	Boil–Spin DNA Isolation and In-House qPCR	Abbott Architect qHBsAg	In-House qHBsAg (1:10 Dilution)	Abbott Architect HBeAg	In-House qHBeAg (1:10 Dilution)
1	B	2.18	1.17	1.93	n.d.	2.9	3.3	-	n.d.
2	D	8.18	6.49	4.99	5.16	5.1	3.7	+	3.68
3	A	2.78	3.44	1.55	n.d.	3.5	3.5	-	n.d.
4	B	5.58	4.70	3.52	2.94	2.9	3.4	-	n.d.
5	C	2.78	3.33	1.38	2.49	3.2	3.6	-	n.d.
6	C	2.68	3.05	2.59	n.d.	3.4	3.7	-	n.d.
7	E	3.08	3.39	2.47	2.86	2.8	3.0	-	n.d.
8	E	2.98	3.61	2.08	1.91	2.2	2.9	-	n.d.
9	E	3.18	3.25	2.41	2.02	3.7	3.6	-	n.d.
10	D	2.08	2.51	2.01	2.05	4.1	3.6	-	n.d.
11	C	0.98	0.88	0.91	2.62	n.d.	n.d.	-	n.d.
12	B	7.98	7.68	5.82	6.82	4.8	3.7	+	3.53
13	C	2.58	2.58	n.d.	n.d.	4	3.7	-	n.d.
14	A	2.58	2.58	2.66	2.23	3.2	3.5	-	n.d.
15	E	3.38	3.82	2.27	2.89	3	2.8	-	n.d.
16	D	8.78	7.55	5.89	6.33	5	3.6	-	3.70
17	D	2.18	3.01	2.18	n.d.	2.5	3.0	-	n.d.
18	B	4.88	4.83	1.82	4.17	3.3	3.6	-	n.d.
19	B	1.68	2.39	1.76	n.d.	2.3	3.5	-	n.d.
20	C	8.78	8.05	6.45	7.48	4.3	3.7	+	3.15
21	B	1.78	1.99	2.12	2.34	1.9	2.5	-	n.d.
22	B	4.18	3.49	2.66	n.d.	3.7	3.7	-	n.d.
23	D	1.48	1.95	1.01	n.d.	4	3.6	-	n.d.
24	C	8.48	7.82	5.94	6.70	4.4	3.2	+	3.67
25	C	2.88	3.23	2.38	2.38	2.5	2.0	-	n.d.
26	C	2.18	2.48	n.d.	n.d.	3.3	2.7	-	n.d.
27	B	0.98	3.18	n.d.	n.d.	2.5	2.9	-	n.d.
28	A	4.48	5.32	2.04	3.86	4	3.6	-	n.d.
29	D	3.08	3.16	2.31	1.74	3.8	3.5	-	n.d.
30	B	7.88	7.86	5.70	6.62	4.2	3.7	+	3.65
31	B	0.98	2.36	n.d.	n.d.	n.d.	n.d.	-	n.d.
32	C	6.08	6.12	3.99	4.94	4.4	3.7	+	3.63
33	C	4.15	4.26	2.39	3.20	2.9	3.3	-	n.d.
34	C	5.49	5.58	3.72	4.76	3.5	3.6	-	n.d.
35	F	6.43	4.83	2.93	3.34	4.74	3.6	+	3.55

**Table 2:** Comparison of in-house assays to clinical diagnostic and point-of-care assays for detection of HBsAg, HBeAg, and HBV DNA.

Marker Detection	Technology	Detection Method	Turnaround Time	Limit of Detection	Sample Volume	Result Type	Cost/Sample	Ref.
HBsAg	Abbott Architect HBsAg qualitative	chemiluminescence ELISA	1 h	0.041 IU/mL	10–150 µL	qualitative	USD $25 *	[14,15]
	Abbott Architect HBsAg quantitative	chemiluminescence ELISA	1 h	0.041 IU/mL	10–150 µL	quantitative	USD $400 *	[14,15]
	Lateral flow	colorimetric	30 min	0.1–2 IU/mL	50 µL	qualitative	USD $0.4–2.8	[33]
	In-house ELISA	chemiluminescence ELISA	4–24 h	0.78 IU/mL	2–20 µL	quantitative	CAD $0.18–0.20	Table S1
HBeAg	Abbott Architect	chemiluminescence ELISA	1 h	0.5 PEI U/mL	10–150 µL	qualitative	USD $65 *	[14,15,34]
	Lateral flow assay	colorimetric	30 min	316 PEI U/mL	50 µL	qualitative	USD $0.4–2.8	[13]
	In-house ELISA	chemiluminescence ELISA	4–24 h	0.39 PEI U/mL	1 µL	quantitative	CAD $0.20	Table S1
HBV DNA	Abbott Architect	qPCR	1 h	10 HBV copies IU/mL	0.5–1 mL	quantitative	USD $325 *	[14,35]
	Silica column + in-house qPCR	qPCR	3.5 h	1.8 × 10^3^ HBV copies IU/mL	50 µL	quantitative	CAD $4.9	Tables S2 and S4
	Column-free DNA extraction (patient plasma) + qPCR	qPCR	3 h	1.8 × 10^4^ HBV copies IU/mL	50 µL	quantitative	CAD $0.49	Tables S3 and S4
	Boil–spin + qPCR	qPCR	2 h 15 min	>2.0 × 10^4^ HBV copies IU/mL	50 µL	quantitative	CAD $0.43	[18]
	Column-free DNA extraction (cultured cells) + qPCR	qPCR	3 h	1.8 × 10^4^ HBV copies IU/mL	cells in 96-well plate	quantitative	CAD $0.48	Tables S3 and S4
	Direct HBV quantification from supernatant of cultured cells	qPCR	2 h	1.8 × 10^4^ HBV copies IU/mL	cells in 96-well plate	quantitative	CAD $0.43	Tables S3 and S4

Costs inclusive of labor and reagents were provided by Alberta Precision Laboratories and included in Supplemental Table S5. * Assay costs for commercial Abbott HBsAg, HBeAg, and HBV DNA assays were obtained from [15], which were provided by Quest Diagnostics and are inclusive of reagents and labor.

## Data Availability

The data that support the findings of this study will be shared on reasonable request to the corresponding author.

## Supplementary Materials

The following supporting information can be downloaded at: https://www.mdpi.com/article/10.3390/pathogens14030258/s1. Figure S1: HBsAg dot blot antibody testing using Ftizgerald antibodies; Figure S2: HBsAg dot blot antibody testing using Fitzgerald and Thermofisher antibodies; Table S1: Cost determination of reagents for HBsAg and HBeAg ELISA; Table S2: Cost determination of reagents for Qiagen DNeasy Blood and Tissue Kit; Table S3: Cost determination of reagents for column-free DNA extraction; Table S4: Cost determination of reagents required for 96-well plate qPCR. Table S5: Cost of HBV diagnostic assays, including overhead and labor, provided by Alberta Precision Laboratories in Alberta, Canada.

## Abbreviations

The following abbreviations are used in this manuscript:

CAD	Canadian dollars
cccDNA	Covalently closed circular DNA
CHB	Chronic hepatitis B
CHREB	Conjoint Health Research Ethics Board
EC_50_	Half-maximal effective concentration
ELISA	Enzyme linked immunosorbent assay
HBeAg	Hepatitis B e antigen
HBsAg	Hepatitis B surface antigen
HBV	Hepatitis B virus
IU/mL	International units per milliliter
LLOD	Lower limit of detection
OD	Optical density
PCR	Polymerase chain reaction
PEI U/mL	Paul Ehrlich Institute units per milliliter
POC	Point of care
qPCR	Quantitative polymerase chain reaction
RLS	Resource-limited setting
TDF	Tenofovir disoproxil fumarate
