# Supplementary material for: Development of Low-Cost In-House Assays for Quantitative Detection of HBsAg, HBeAg, and HBV DNA to Enhance Hepatitis B Virus Diagnostics and Antiviral Screening in Resource-Limited Settings
Source: Pathogens. 2025 Mar 5;14(3):258. doi: 10.3390/pathogens14030258 (PMC11945746; doi:10.3390/pathogens14030258)
Supplement: Supplementary file 1 [file pathogens-14-00258-s001.zip › pathogens-3471370-supplementary.pdf]

Supplemental Document

Development of Low-Cost In-House Assays for Quantitative Detection of HBsAg, HBeAg, and HBV DNA to Enhance Hepatitis B Virus Diagnostics and Antiviral Screening in Resource-Limited Settings

Simmone D'souza [1,2,3], Layla Al-Yasiri [1,2], Annie Chen [1,2], Dan T. Boghici [1,2],

Guido van Marle [1], Jennifer A. Corcoran [1], Trushar R. Patel [1,3] and Carla S. Coffin [1,2,*]

1 Department of Microbiology, Immunology and Infectious Diseases, Cumming School of Medicine, University of Calgary, Calgary, AB T2N 2T8, Canada; simmone.dsouza@ucalgary.ca (S.D.); layla.alyasiri1@ucalgary.ca (L.A.-Y.); annie.chen2@ucalgary.ca (A.C.); dan.boghici@mail.mcgill.ca (D.T.B.); vanmarle@ucalgary.ca (G.v.M.); jennifer.corcoran@ucalgary.ca (J.A.C.); trushar.patel@uleth.ca (T.R.P.)

2 Department of Medicine, Cumming School of Medicine, University of Calgary, Calgary, AB T2N 2T8, Canada

3 Department of Chemistry and Biochemistry, Alberta RNA Research and Training Institute, University of Lethbridge, Lethbridge, AB T1K 3M4, Canada

* Correspondence: cscoffin@ucalgary.ca

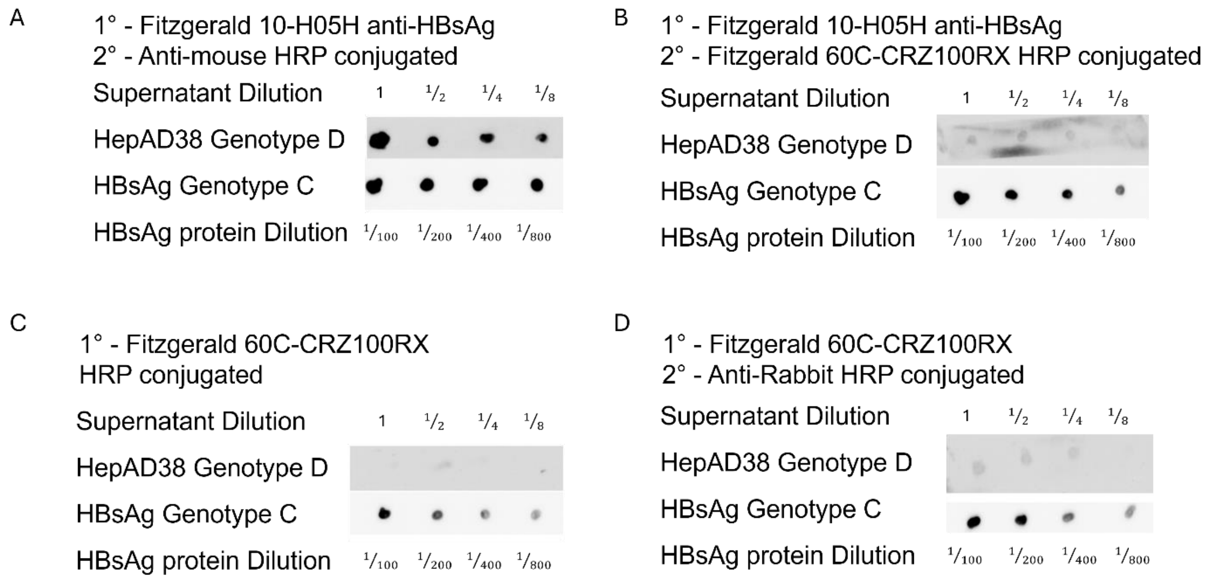

Figure S1. Dot blot hybridization testing a variety of anti-HBsAg antibodies against HBsAg protein standard samples (Fitzgerald C-CP2019R) at 1/100, 1/200, 1/400 and 1/800 dilutions and HepAD38 supernatant samples at serial dilutions (1, $^{1}/_{2}$, $^{1}/_{4}$, $^{1}/_{8}$). The test results included: (A) the primary HBsAg antibody (Fitzgerald 10-H05H) with anti-mouse HRP-conjugated antibody, (B) the combination of primary HBsAg antibody (Fitzgerald 10-H05H) and the secondary HBsAg antibody (Fitzgerald 60C-CRZ100RX HRP-conjugated) (C) the secondary HBsAg antibody (Fitzgerald 60C-CRZ100RX HRP-conjugated) alone, and (D) the secondary HBsAg antibody (Fitzgerald 60C-CRZ100RX) with an anti-rabbit HRP-conjugated antibody.

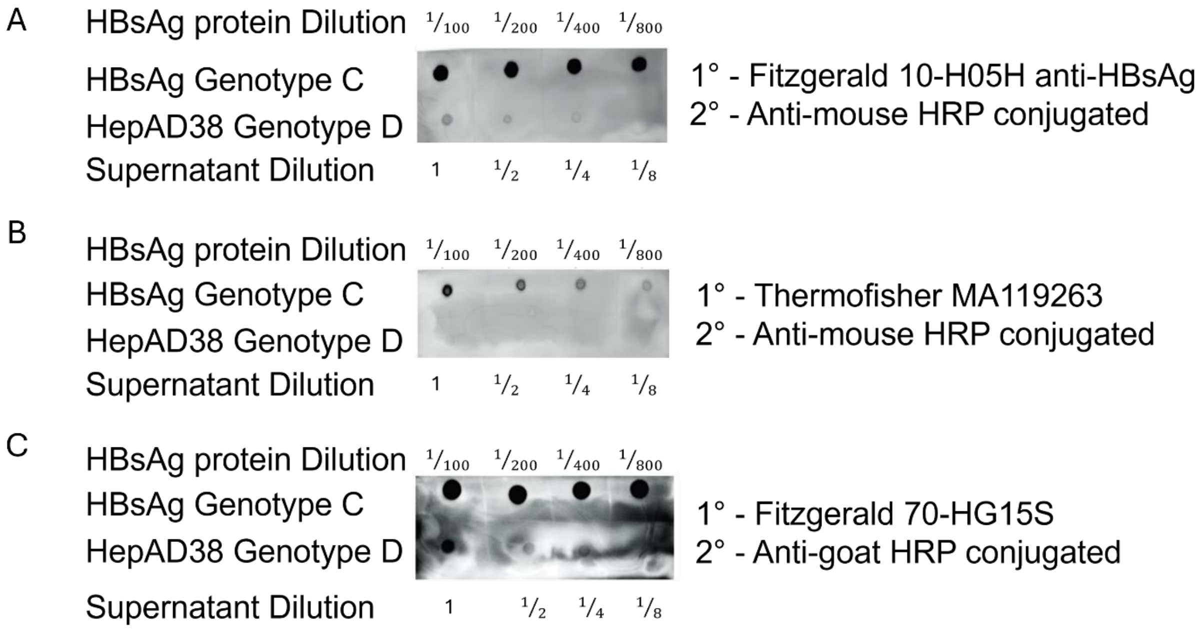

Figure S2. Protein dot blot testing a variety of anti-HBsAg antibodies against HBsAg protein standard samples (Fitzgerald C-CP2019R) at 1/100, 1/200, 1/400 and 1/800 dilutions and HepAD38 supernatant samples at 1, $^{1}/_{2}$, $^{1}/_{4}$, and $^{1}/_{8}$ serial dilutions. The results included (A) 1:2000 diluted primary HBsAg antibody (Fitzgerald 10-H05H) with anti-mouse HRP-conjugated antibody for visualization, (B)1:2000 diluted HBsAg antibody (Thermo Fisher MA119263) and an anti-mouse HRP-conjugated antibody, and (C) 1:5000 diluted secondary HBsAg antibody (Fitzgerald 70-HG15S) with an anti-goat HRP-conjugated antibody.

Table S1. Cost determination in Canadian Dollars ($CAD) of reagents needed for quantification of HBsAg or HBeAg ELISA in a single 96-well plate.

Reagents	Supplier	Catalog #	Quantity supplied	Upfront reagent Cost ($CAD)	Amount of stock used in ELISA	HBsAg ($CAD)	HBeAg ($CAD)
**96-well high-binding microtiter plates**	Millipore Sigma	CLS9018-100EA	100 plates	$627	1 plate	$6.3	$6.3
**α-HBsAg mouse monoclonal antibody (Coating antibody)**	Fitzgerald / Biosynth	10-H05H	1mg	$238	9.6ug	$2.3	$2.3
**α-HBsAg goat polyclonal antibody (secondary antibody)**	Fitzgerald / Biosynth	70-HG15S	1mg	$220	see below	see below	N/A
**Lynx Rapid HRP Antibody Conjugation Kit (used for conjugating HRP to 70-HG15S)**	Biorad	LNK002P	(3 conjugations for 400ug antibody each)	$710			
**LNK002P conjugated to HRP**	Fitzgerald / Biosynth +Biorad	70-HG15S + LNK002P	1mg	$930	9.6ng	$0.009	N/A
**α-HBeAg mouse monoclonal antibody (Coating antibody)**	Fitzgerald / Biosynth	10-H10M	1mg	$150	9.6ug	N/A	$1.44
**α-HBeAg mouse monoclonal antibody (detection antibody)**	Fitzgerald / Biosynth	61-H10K	1mg	$172	9.6ng		$0.002
**HBsAg (protein standard)**	Fitzgerald / Biosynth	C-CP2019R	1mg	$600	50ng	$0.03	N/A
**HBeAg (protein standard)**	Fitzgerald / Biosynth	30-AH18	1mg	$1750	50ng	N/A	$0.09
**TMB (3,3',5,5'-Tetramethylbenzidine) chromogenic substrate**	ThermoFisher Scientific	00-2023	500mL	$274	9.6mL	$5.26	$5.26

**Hydrochloric Acid (HCl)**	Sigma-Aldrich	320331-500mL	500mL	$111	0.8mL	$0.18	$0.18
**Na_2CO_3**	Sigma-Aldrich	223530-500G	500g	$118	0.017g	$0.004	$0.004
**$NaHCO_3$**	Sigma-Aldrich	S6014-1KG	1kg	$130	0.029g	$0.004	$0.004
**PBS**	Sigma-Aldrich	P4417-100TAB	100 tab = 20L PBS	$150.4	100mL	$0.75	$0.75
**Tween-20**	Sigma-Aldrich	P2287-500mL	500mL	$82.5	2mL	$0.33	$0.33
**Bovine Serum Albumin Fraction V**	Roche	3117332001 (50g)	50g	$229	0.5g	$2.29	$2.29
**Cost summary ($CAD)**				$5561.9		$17.4	$18.9

Table S2. Cost determination in Canadian Dollars ($CAD) of reagents for Qiagen DNeasy Blood and Tissue Kit [25] .

**Reagents**	**Supplier**	**Catalog #**	**Quantity supplied**	**Upfront reagent cost ($CAD)**	**Reagent amount used for single patient**	**Cost for single patient sample ($CAD)**
**Qiagen DNeasy Blood and Tissue Kit**	Qiagen	69504	50 columns	$224	1	$4.48

Table S3. Cost determination in Canadian Dollars ($CAD) of reagents for column-free DNA extraction of a single plasma sample or 96-well plate HepAD38 cells.

Reagents	Supplier	Catalog #	Quantity supplied	Upfront reagent cost ($CAD)	Reagent amount used for single patient	Cost for single patient sample ($CAD)	Reagent amount used for 96-well plate cell processing	Cost for 96-well plate ($CAD)
**96-well plates**	BioBasic	PCR-960-LP-S2	25 plates	$108.39	N/A	N/A	1 plate	$4.34
**1.5mL tubes**	BioBasic	BT620-NS	1000 tubes	$37.91	1 tube	$0.04	N/A	N/A
**Proteinase K**	BioBasic	401.SIZE.5ml	5mL of 20mg/mL	$72.24	30ug	$0.02	960ug	$0.69
**Tris-HCl**	Sigma Aldrich	T15760-500G	500g	$161	1.8ug	$0.0006	5.8mg	$0.002
**EDTA**	Sigma Aldrich	E8008-100ML	100mL	$27.5	1.5uL	$0.0004	48uL	$0.01
**SDS**	Sigma Aldrich	75746-250G	250g	$90.8	0.3ug	$0.0001	9.6ug	$0.003
**NaCl**	Sigma Aldrich	S9888-1KG	1kg	$116	1.75ug	$0.0002	55ug	$0.06
**NP-40**	Sigma Aldrich	492016-100ML	100mL	$96.2	0.38uL	$0.0004	12uL	$0.01
**Cost summary ($CAD)**				$710.04		$0.06		$5.1

Table S4. Cost determination in Canadian Dollars ($CAD) of reagents required for 96-well plate quantitative (q)PCR.

**Reagents**	**Supplier**	**Catalog #**	**Quantity supplied**	**Upfront reagent Cost ($CAD)**	**cost per plate ($CAD)**	**cost per well ($CAD)**
**HBV 2270F Primer**	IDT	100nmol	1000uL of 10uM	$5.89	$0.28	$0.003
**HBV 2392R Primer**	IDT	100nmol	1000uL of 10uM	$5.89	$0.28	$0.003
**iTaq™ Universal SYBR® Green Supermix, 2,500 x 20 µl rxns, 25 ml (5 x 5 ml)**	Biorad	1725124	25mL	$1323	$25.40	$0.26
**96-well plate**	VWR	82006-650	100	$1129.27	$11.29	$0.12
**Plate seal**	VWR	60941-078	100	$376.78	$3.77	$0.04
**Cost summary ($CAD)**				$2840.83	$41.03	$0.43

Table S5. Cost of HBV Diagnostic Assays, Including Overhead and Labor, Provided by Alberta Precision Laboratories in Alberta, Canada (https://www.albertaprecisionlabs.ca/)

**Marker Detection**	**Assay Name**	**Cost CAD/sample**
HBsAg	Abbott Architect HBsAg qualitative	33.48
	Abbott Architect HBsAg quantitative	39.49
HBeAg	Abbott Architect HBeAg qualitative	39.49
HBV DNA	Abbott Architect RealTime HBV Viral Load Assay	135.03
